# A Narrative Review of the Role of Diet and Lifestyle Factors in the Development and Prevention of Endometrial Cancer

**DOI:** 10.3390/cancers13092149

**Published:** 2021-04-29

**Authors:** Hajar Ku Yasin, Anthony H. Taylor, Thangesweran Ayakannu

**Affiliations:** 1 Department of Obstetrics & Gynaecology, Cumberland Infirmary, Carlisle CA2 7HY, UK; hajar.kuyasin@nhs.net; 2 Department of Molecular and Cell Biology, University of Leicester, Leicester LE1 7RH, UK; aht13@leicester.ac.uk; 3 Gynaecology Oncology Cancer Centre, Liverpool Women’s NHS Foundation Trust, Liverpool Women’s Hospital, Liverpool L8 7SS, UK

**Keywords:** endometrial cancer, diet, nutrients, hormones, diabetes, lipids, melatonin, obesity, vitamins, minerals

## Abstract

**Simple Summary:**

The incidence and prevalence of endometrial cancer is increasing globally. The main factors involved in this increase have been the way women live today and what they eat and drink. In fact, the obesity pandemic that is sweeping across the planet is considered to be the main contributory feature. This review aims to introduce to a new audience, those that are not experts in the field, what is known about the different types of endometrial cancer and the mechanisms for their induction and protection. We also seek to summarise the existing knowledge on dietary and lifestyle factors that prevent endometrial development in susceptible populations and identify the main problem in this arena; the paucity of research studies and clinical trials that investigate the interaction(s) between diet, lifestyle and endometrial cancer risk whilst highlighting those areas of promise that should be further investigated.

**Abstract:**

Endometrial cancer is the most common cancer affecting the reproductive organs of women living in higher-income countries. Apart from hormonal influences and genetic predisposition, obesity and metabolic syndrome are increasingly recognised as major factors in endometrial cancer risk, due to changes in lifestyle and diet, whereby high glycaemic index and lipid deposition are prevalent. This is especially true in countries where micronutrients, such as vitamins and minerals are exchanged for high calorific diets and a sedentary lifestyle. In this review, we will survey the currently known lifestyle factors, dietary requirements and hormonal changes that increase an individual’s risk for endometrial cancer and discuss their relevance for clinical management. We also examine the evidence that everyday factors and clinical interventions have on reducing that risk, such that informed healthy choices can be made. In this narrative review, we thus summarise the dietary and lifestyle factors that promote and prevent the incidence of endometrial cancer.

## 1. Introduction

What people eat (dietary patterns) and how they live (lifestyle habits) are the main modifiable risk factors associated with the development of cancer [1]. Keeping a healthy body weight, eating the right foods and undertaking regular exercise are considered important for not only cardiovascular health, but also for cancer prevention. This conclusion has come from many research studies, reviews and government information sources over the past 20 years or so, and have provided a consensus that cessation of smoking, reduced alcohol intake and having a healthy lifestyle are predominantly important in reducing cancer risk [2,3,4]. Some reports have indicated that psychological stress may be a contributory factor in the development of cancer [5], but such data is scarce and inconclusive. Such stressors may impact on lifestyle and diet, which in turn may impact on cancer development [6]. Currently there is no convincing data to suggest stress is an independent factor in the development of EC [7]. Nevertheless, there are many other factors that increase the risk of developing EC. In particular, data has emerged that the storage of fats and lipids may exacerbate any genetic predisposition towards the development of cancer [8,9]. Despite this knowledge, it has been estimated that at least 20% of all women worldwide will die from cancer alone by 2050, primarily due to the increasing incidence of obesity, which is now in pandemic proportions [10].

**Figure 1:**
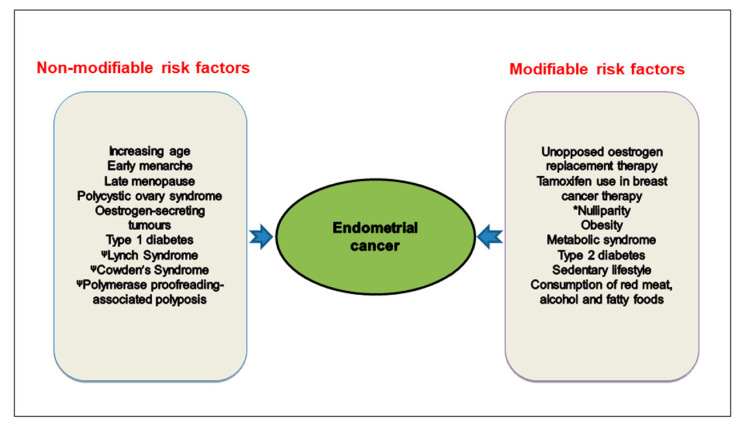
Epidemiological, genetic, clinical, lifestyle and dietary risk factors for the development of endometrial cancers. Two distinct pathways are depicted in which non-modifiable and modifiable risk factors are listed. * Nulliparity is included as modifiable risk factor because pregnancy can reduce the risk of EC development. It could also conceivably be included as a non-modifiable risk factor, since some women are unable to conceive. ^ψ^ Familial genetic mutations are also risk factors for EC and are associated with Lynch syndrome, Cowden’s syndrome, and Polymerase proof-reading polyposis (see Figure 5).

For cancers in women, this is of particular importance because oestrogen generated from the fats and lipids stored in adipose tissue or produced by the adrenal cortex are known to be associated with breast, ovarian and endometrial cancer (EC) [11,12,13,14]. Many studies have suggested that healthy dietary patterns and good lifestyle habits may also decrease the risk of developing other cancers prevalent in women, such as colorectal, pancreatic and thyroid [15,16,17,18,19,20]. Of all the female-associated cancers, those of the breast, cervix, ovary and endometrium are foremost in women’s minds, because they often know of close family members or friends that have been diagnosed (or have died) with these female-specific cancers. Despite improved overall survival rates for breast, ovarian and endometrial cancers over the past 20 years [21,22], the incidence of EC has increased by 40% and the mortality rate for women with EC has risen by 20% in the last decade [23]. This increase in the incidence of EC has been suggested to be caused by two key factors; (1) an increase in obesity in women and (2) an ageing female population. Although these are prominent risk factors, EC is a multifactorial disease, with exposure to excess oestrogen and/or a relative lack of progesterone [24,25,26] being the biggest stimulator. This is because oestrogen stimulates rapid growth of endometrial cells, whereas progesterone opposes this action. Long-term treatment of menopausal symptoms with conventional hormone replacement therapy consisting of unopposed oestrogen (oestrogen without a protective progestogen) leads to an increased risk of developing EC [24,27,28,29]. Additionally, other risk factors that have been associated with EC include diabetes, and diets high in sugar, animal fats, and cholesterol (Figure 1) [30,31,32,33,34,35].

This review will focus on the underlying mechanism(s) for the development of EC in relation to these factors, dietary patterns and lifestyle habits [36,37,38,39], with numerous studies explored to provide evidence of an association between individual nutrients or foods and EC risk. What is important to a patient worried about developing cancer, is not only what lifestyle and dietary choices should be avoided, but also what should be encompassed and so lessen the risk of developing the disease in the first place, or what foods and lifestyle adaptations should be adopted once the disease has been diagnosed. It is our contention that an examination of existing dietary patterns and their association with EC risk could provide a wider and more realistic estimation of possible relationships between food and healthy habits in a particular female population. This knowledge may provide useful areas for future research into an increasingly prevalent public health issue for our ageing female population.

### Methods

A literature search of the Embase, Medline, ClinicalKey, CINAHL and PubMed databases was conducted (from 1974 to May 2020) with the following search terms (*“endometrium cancer”/OR *“endometrium carcinoma”/OR *“endometrium sarcoma”/OR (endometri* ADJ2 cancer*/OR (“endometrial neoplasms”/OR (*“carcinoma, endometrioid).ti,ab) AND (*“Diet”/OR (“lifestyle*”).ti,ab)) [Publication types Article OR Conference Paper OR Journal OR Review] [English language] [Female] [Human age groups Adult 18 to 64 years OR Aged 65+ years] [Humans]’ and yielded 523 PubMed/Embase results and 579 Medline results. Duplicate or irrelevant references were omitted revealing 154 directly applicable citations. The Cochrane systematic reviews database was also searched using the search terms “heathy lifestyle index” and “endometrial cancers” and generated three articles (one from 2015 and two from 2018). The reference lists of these were also scanned, aided by citations provided by the article reviewers and additional references obtained. Only literature supportive of the statements used are provided.

## 2. Endometrial Cancer Incidence and Prevalence

EC is the 4th most common cancer affecting women in the United Kingdom, accounting for 5% of all new cases annually. It is the 8th most common cause of death in women in the UK, with 34% of the EC cases considered to be preventable [23]. It is mainly a disease of high-income countries, with the highest incidences occurring in Central and Eastern Europe, North America and Northern Europe (Table 1).

**Figure 2:**
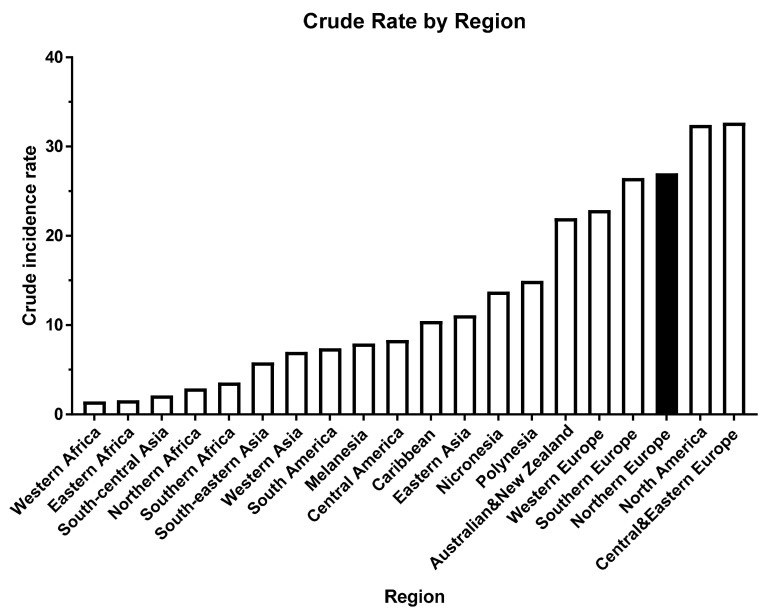
Estimated crude incidence rate for endometrial cancer (all ages; 0–85+) in 2018, based on different regions of the world. Data are shown from lowest incidence rate to highest incidence rates with Northern Europe highlighted with the solid bar. Adapted from [40].

Although EC incidence has historically been much lower in Asia, Africa and South America (Figure 2) [40,41,42], the economic boom in these continents over the past decade has allowed a dietary change towards a more Westernised diet, even when age is taken into account [40,41]. This is an issue that can be directly attributable to a change in diet/lifestyle and in some cases to an increased production of circulating oestrogens [43,44,45] or the use of unopposed oestrogen therapy for menopausal symptoms, where the risk of EC increases by up to 70-fold; adding a progestogen reduces the risk to that of the general population [46].

It is also important to note that income is also related to EC incidence prevalence and hence mortality rates (Table 1), with women from more affluent backgrounds having higher values of these endpoints than women from less affluent backgrounds. This again is attributed to easier access to high calorific foods and increased risk of obesity [47,48]. Fat stored in adipose tissues can generate oestrogen precursors that drive endometrial hyperplasia and carcinoma [32,34,35,49,50,51,52,53]. Some studies have shown that as many as 40% of EC cases may be attributable to this [54]. Weighing more than 200 lbs (~90 kg or ~14 stones) increases risk by about 7-fold [46]. In a 2007 analysis of data on 1.2 million women, each 10-unit increment in body mass index (BMI) was associated with a nearly 3-fold increase in EC risk [55]. Other mechanisms by which obesity may increase EC risk include perturbation of glucose regulation and promotion of an inflammatory state throughout the body [25,56]. A recent study of 33,436 postmenopausal EC cases confirmed that BMI was a strong predictor of EC risk (RR 4.41; 95% CI 2.7–7.2 for BMI ≥ 35 kg/m^2^), but this association was no longer evident among users who had previously used HRT [57]. These data need to be examined in detail, since menopausal status and histological subtypes had no bearing on the relative risk calculations [58]. This means that it did not matter if a woman was postmenopausal or what histologically defined type of EC she was diagnosed with, her risk of developing EC was strongly positively correlated with her BMI at the time of diagnosis, with the relative risk for each study being 20.7 when BMI was ≥42 kg/m^2^ or 4.41 when BMI was ≥35 kg/m^2^, respectively [58] and that previous exposure to the hormones in HRT either completely or partially negated this effect. These data suggest that residual drug deposition has an on-going interaction with the control of BMI and subsequent reduction in EC risk. Support for these observations comes from an older cohort study of 103,882 postmenopausal women where different forms of HRT significantly modified the relationship between BMI and EC risk [59].

## 3. Types of Endometrial Cancer

EC can be categorised based on cellular morphology and where the malignant cells are situated within the uterus or outside this organ (metastatic disease/secondary tumour/recurrent disease). The major division of EC types is based on tissue morphology of which endometrioid adenocarcinoma, clear cell carcinoma, mixed cell carcinoma, mucinous carcinoma, serous carcinoma, squamous carcinoma, undifferentiated carcinoma and metastatic carcinoma are the major types (Table 2). Endometrioid adenocarcinoma, also described as type 1, is the most common type of EC and accounts for 80–90% of cases [60]. Phenotypically, endometrioid adenocarcinoma is considered to be oestrogen-dependent, in that tumour cell survival is dependent on endogenous or exogenous oestrogen stimulation [54,61]. It is therefore not surprising that exogenous or endogenous oestrogen stimulation results in a higher incidence of type 1 EC. Non-endometrioid, also described as type 2 EC consists of the other subtypes of EC in the list above, excluding endometrioid adenocarcinoma, with serous carcinoma (~10%) and clear cell carcinoma (~3%) being the most prevalent [62]. The other type 2 EC types (metastatic, mixed cell, undifferentiated, squamous and mucinous types are rarer forms and make up the remaining percentages) [63]. The major difference between type 1 and type 2 EC is that type 2 (non-endometrioid) forms of malignancy do not require oestrogen stimulation for cell survival due to gene mutations [64]. Furthermore, when considering both biological and clinical parameters, gene mutations are currently used for EC classification, whereby type 1 carcinomas that comprise 80–90% of newly-diagnosed EC, have alterations in the phosphatase and tensin homolog gene (*PTEN*), the Kirsten ras gene (*KRAS*), the beta-catenin gene (*CTNNB1*) and DNA characterised by microsatellite instability (MSI) [65,66]. These tumours are associated with better prognosis [65,66]. Whereas, in type 2 EC tumours are defined in this classification system as having *TP53* mutations, a high Ki-67 (*MIB1*) score (a marker of proliferation), tumour suppressor gene p16 inactivation, increased cadherin-1 gene (*CDH1*) expression and the amplification of human epidermal growth factor receptor 2 gene (*HER2*), which is associated with metastatic disease and tumour spread [67,68].

**Figure 3:**
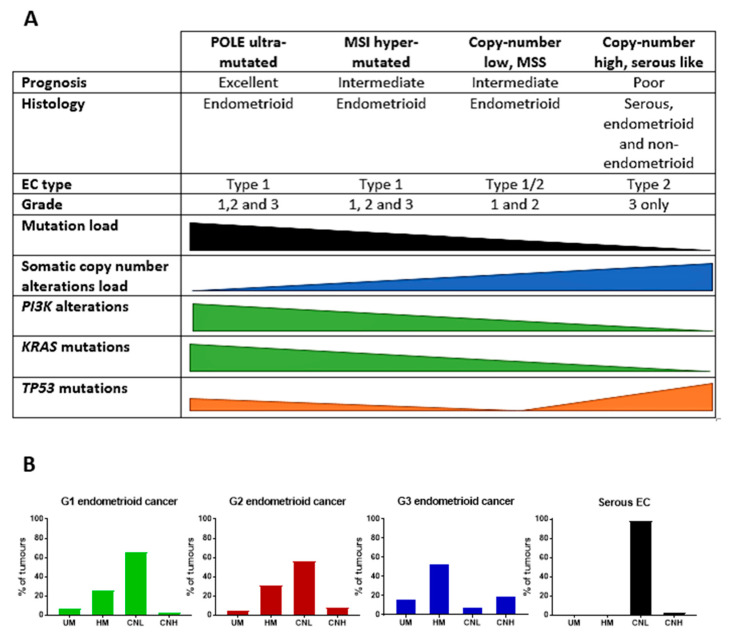
Overview of the molecular classification systems for EC. Panel (**A**) indicates that genetic mutations are related to the four genomic classes of EC, defined as the Cancer Genome Atlas (TCGA) classification through *POLE* mutations, which are ultramutated (UM); Multisatellite (MSI) instability events, which are hypermutated events (HM); or are copy number low (CNL) or copy number high (CNH). The relationships between these four genomic classes and patient prognosis, pathognomic factors and other gene alterations is depicted. In Panel (**B**), the distributions of these TCGA genomic classes is shown as a percentage of the tumours based on the trialistic grading system and with respect the Type 2 serous EC. The % of tumours that have ultramutated *POLE* mutations (UM); hypermutated MSI (HM); or are copy number low (CNL) or copy number high (CNH) are shown. Where the bar is not visible, the data indicate that none of those tumours have that event associated with them. Data are adapted from [70,71].

In addition, in order to provide a more accurate classification for prognostic purposes, clinicopathological information and gene mutation data have been integrated into a more modern classification system [69]. This newer system performed by The Cancer Genome Atlas (TCGA) consortium provides four prognostic subgroups, with the *POLE* group having the best prognosis (Figure 3A) [62,63,70,71]. The tumours here have *POLE* exonuclease domain mutations, which is part of the catalytic unit of DNA polymerase. The microsatellite instability (MSI) group, composed of patients with EC lesions having DNA microsatellite instability have the next worst prognosis, followed by the Copy Number Low (CNL) and Copy Number High (CNH) groups (Figure 3B). Copy number groups are defined by a differential profile of copy alterations (CNA), with the CNH group particularly presenting an elevated incidence of tumour suppressor p53 (*TP53*) mutations and worse prognosis when compared to the low copy number group [70]. Linking these four molecular tumour subgroups to existing FIGO classifications is problematic, and not straight-forward since there is some overlap in these molecular markers for type 1 and type 2 EC [62,71], but in general *TP53* mutations are more frequently associated with type 2 EC [serous carcinoma (59–93%), carcinosarcoma (44–91%) and clear cell carcinoma (28–46%)] than with type 1 EC (5–14%) (Figure 3B) [71]. By contrast, *PTEN* mutations are associated more with type 1 EC (64–80%), than with type 2 EC (serous carcinoma (2–3%), carcinosarcoma (11–33%) and clear cell carcinoma (0–21%)). Details on other TCGA gene mutations (and others not mentioned herein can be found in a recent review on this subject and references therein [71]) and their relative frequencies are shown in Table 3. The effect of tumour grading in type 1 EC can also be of use in designing treatment strategies [71] but has also come under scrutiny and remains controversial when molecular profiling is applied because tumour classification itself is complicated when stage and grade are applied [72,73]. Obviously, more work on how to best classify endometrial cancer types and subtypes for risk prevention, diagnosis, prognosis and treatment is required, but beyond the scope of the current review.

## 4. Mechanisms of Oestrogen-Induced Endometrial Cancer

**Figure 4:**
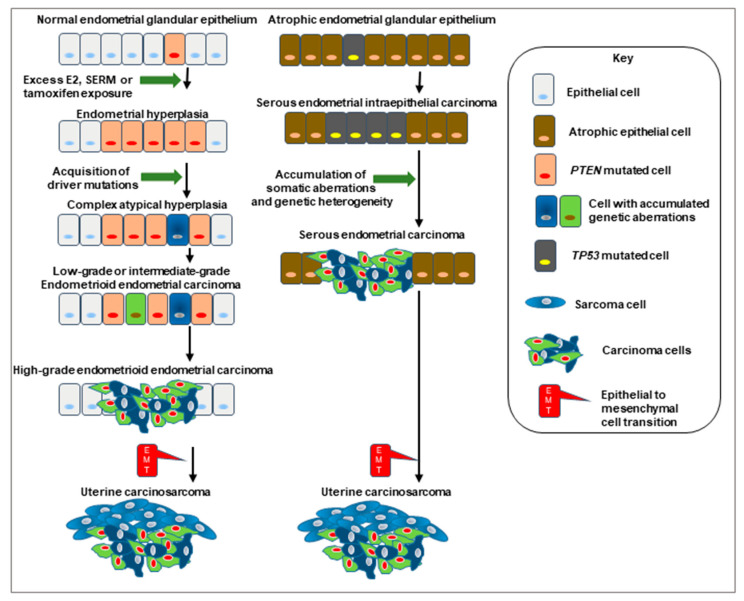
Overview of endometrial cancer origin and development. A schematic representations of the development of and progression of endometrioid (Type 1) EC (left side) and of non-endometrioid serous (Type 2) EC (right side) from their respective normal and atrophic endometrial glandular epithelial cells. During initiation, *PTEN* or *TP53* gene mutations (early events in the aetiopathology of many endometrioid and serous ECs) and other genetic aberrations result in the formation of precursor lesions (complex atypical hyperplasia and serous intratumoural intraepithelial carcinoma, respectively). The epithelial cells that accumulate mutations and genetic alterations are coloured. Sometimes, carcinomas (particularly high-grade tumours) undergo epithelial to mesenchymal cell transition (EMT), giving rise to uterine carcinosarcomas, which as dualistic tumours consist of both epithelial carcinoma cells and sarcoma cells (dark blue and green cells and ovoid blue cells, respectively). Data are adapted from [70,71].

Firstly, it is known that long-lasting and widespread use of unopposed exogenous oestrogen therapy by postmenopausal women for the treatment of perimenopausal and postmenopausal symptoms (such as vasomotor signs, mood swings, etc.) increases the risk of developing endometrial pathologies, such as epithelial cell hyperplasia, cystic gland formation, polyp formation, adenomyosis, leiomyomata and in some cases these pathologies converge into type 1 EC in susceptible women [84,85]. Many of these pathological changes can be mimicked by the adjuvant use of tamoxifen in women with primary breast cancer and are attributed to the direct action of the oestrogens and tamoxifen at the oestrogen receptor to alter cell proliferation and reduce the actions of pro-apoptotic signalling (Figure 4) [86].

Secondly, extended longevity, resulting in a higher median age for women, results in a promulgation of these effects such that DNA or oxidative damage and other DNA/RNA/ miRNA mutational events accumulate in affected cells and so create the focus for endometrial hyperplasia that is a precursor for the early stage development of EC [87,88]. This early stage EC (type 1, grade 1) is particularly difficult to detect, as such patients are often asymptomatic.

A third factor is a sedentary lifestyle (Figure 1). As women age, the amount of physical activity they partake in reduces [4,89,90]. This reduction in physical activity, can result in a change in body mass, shape and increased deposition of fats and lipids causing obesity [91]. The obesity itself may not be a key determinant in the development of EC, but the increased deposition of lipids in adipose tissue increases the ability of the adipose tissue (especially white fat) to synthesise oestrogens in those peripheral tissues and so increase the levels of circulating oestrogens that can stimulate cells in the uterus [92,93]. In addition, physical activity is thought to decrease the risk for EC because it reduces serum levels of oestradiol by increasing the levels of sex hormone binding globulin (SHBG), the binding protein for oestradiol that is synthesised in the liver. These effects of physical activity may be mediated through prevention of weight gain [90]. More generally, effects on oestrogen metabolism may, at least in part, operate directly, or through decreasing body fat stores [94].

Additionally, a sedentary lifestyle and a daily routine that does not include any physical activity are thought to play a role because increased risk of insulin resistance leading to the development of type 2 diabetes mellitus [95,96]. Type 2 diabetes and the related metabolic syndrome [97,98], are known risk factors for the advancement of EC [99]. It is not only the lack of physical activity that is a contributory factor here, but also the amount of time spent sitting. For example, women who spend the same amount of time being physically active, but actually spend shorter amounts of time sitting, also have reduced risk of developing EC [90,100]. These data suggest that EC risk occurs through insulin-related mechanisms where low levels of energy expenditure [101] and weight gain [102], factors associated with sitting time [101], are causes of EC development. Conversely, sustained moderate physical activity increases basal metabolic rate and maximal oxygen uptake [103], which in the long term, increases metabolic efficiency and capacity, which reduces circulating insulin levels and insulin resistance [104].

A fourth and final contributory factor, is changes in diet that involve altered absorption and reabsorptions of sterols, since they can alter the regulation and metabolism of oestrogens [105]. This effect is prominent in the gall bladder, where most of the conjugation and excretion of oestrogen is controlled [106,107]. Ingestion of sterols, such as 27-hydroxycholesterol and cholesterol, are also dangerous for women susceptible for EC because they can act as exogenous oestrogens [108] or substrates for oestrogen production [109] and increase endometrial epithelial cell proliferation. Carbohydrates also affect nutrient absorption across the GI tract. For example, increased total dietary carbohydrate intake has been shown to have a modest promoting effect in the development of EC, particularly amongst women who have never used HRT [97]. Long-term consumption of a high carbohydrate diet results in hyperinsulinaemia, which in turn increases the bio-availability of insulin-like growth factor 1 (IGF-1) [110], which directly promotes endometrial epithelial cell growth, reduces cell death and stimulates cell division in EC cell lines [54,111,112]. Insulin and IGF-1 are also powerful negative regulators of sex hormone-binding globulin synthesis in vitro and may therefore stimulate EC development [112], because loss of sex hormone-binding globulin increases the availability of circulating free oestrogens. Polycystic ovary syndrome (PCOS), a hormonal-metabolic disorder, has also been shown to promote EC development providing an approximate 5-fold increased risk [46,54]. PCOS causes excessive production of androgens, which can be converted into oestrogens by the actions of P450 aromatase, which can be found in the endometrium [113] and in omental adipose tissue [114]. This results in excess circulating oestrogen concentrations. Moreover, it has been suggested that the androgens themselves, when present in excess, may increase EC risk directly through activation of the androgen receptor, although this has yet to be clearly established [115,116]. The key molecular factor here is the binding of oestrogen to the oestrogen receptor, where it alters the gene expression pattern of the normal endometrial cell to a proliferative phenotype that allows the accumulation of inherent errors of metabolism and DNA replication to be manifest.

## 5. Mechanism of Oestrogen-Independent Endometrial Cancer

Although most women (80–90%) present with endometrioid (oestrogen-dependent) EC, there are a minority that present with the most difficult form of EC to treat; Type 2 EC (oestrogen-independent), which tend to be an adenocarcinoma-like tumour but not driven by oestrogen (Figure 4) [38,74,117,118,119]. These are life-threatening forms of EC [120], that may be a natural progression from endometrioid (Type 1) cancer [22,121], or may have a completely different and distinct aetiopathology [122,123,124]. The underlying mechanism for the development of Type 2 EC is thought to be due to genetic mutation [65,125] of the tumour suppressor gene *TP53* [124,126,127,128,129], the tumour suppressor gene *PTEN* [39] or over-amplification or mutation of *HER2-neu*, which responds to the actions of epidermal growth factor (Table 3) [130,131,132].

Because dietary factors, lifestyle and environmental factors are known to affect p53 function in hepatoma cancer (aflatoxin B and hepatitis B [133,134,135,136]) and in lung and oesophageal cancer (due to tobacco use [88,137]), it is highly likely that infection and tobacco use could precipitate *TP53* mutations in EC too. Furthermore, an increased risk of *TP53* gene mutations in breast cancer patients due to extended alcohol consumption has been reported [138,139,140] and also through increased lycopene/carotenoid intake (found in tomato and pink grapefruit). These two factors are also associated with EC improvement in some studies [141,142,143,144], but not all [145]. Some interesting data on the effects of folate suggest a possible treatment for all of these cancers in that folate intake was found to be associated with reduce risk of breast cancer development in women whose tumours had p53 mutations [138]. The authors suggested a direct causal link between alcohol intake and p53 mutation concluding that increased alcohol consumption inhibits folate uptake from the diet [138]. Since folate protects against *TP53* mutagenesis, then excess alcohol consumption puts women at risk of developing p53 mutations, and that leads directly to the development of breast and other cancers. Another factor that needs consideration is a Western diet, which consists of a high glycaemic index (amount of sugar absorbed into blood over 2 h), red meat and processed food that is known to contribute to colon cancer development, also through a *TP53* missense mutation [146,147]. These data suggest that a Western diet full of carbohydrates, red meat, alcohol and processed food could cause type 2 EC directly through p53 mutations.

Diabetes mellitus and hyperinsulinaemia are associated with EC [148,149,150,151]. Diabetic postmenopausal women are twice as likely to develop EC as their non-diabetic counterparts [152]. In addition, type 2 diabetics often develop insulin resistance, which results in hyperinsulinaemia, a metabolic state that adds additional EC risk [153]. Moreover, a low level of the hormone adiponectin, which may be a surrogate marker for insulin resistance, has also been associated with increased EC risk in some but not all studies [25,154,155]. Similar to what occurs in healthy cells during diabetes and insulin resistance, EC cells develop abnormalities in the insulin and insulin-like growth factor-1 (IGF-1) signalling pathways, both of which are involved in cancer cell growth. Thus, it is not surprising that the anti-diabetic drug metformin, which improves insulin sensitivity, has received considerable attention from researchers investigating new ways to combat EC [25,155,156,157,158]. Metformin is a drug that lowers blood glucose levels by reducing the ability of the liver to produce new glucose from glycogen and also increases the ability of muscle cells to take up glucose from the blood [159,160]. A variety of epidemiological studies have shown that diabetic patients taking metformin are significantly less likely to develop a variety of cancers, including those of the pancreas, liver, colon-rectum, and breast [161,162,163,164,165]. With respect to EC, preclinical studies have shown that metformin inhibits the proliferation and promotes the death of EC cells [156,166,167].

**Figure 5:**
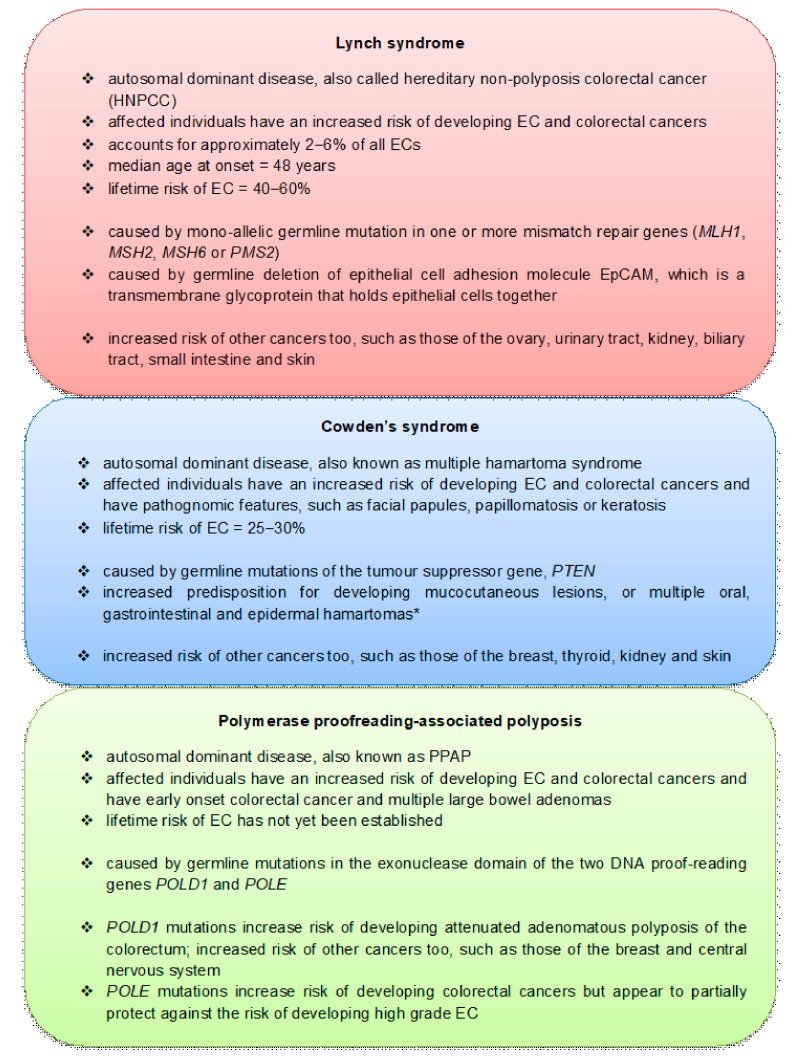
Hereditary syndromes manifesting as increased risk of developing EC. Each of these diseases is inherited in an autosomal dominant manner and each is a non-modifiable risk factor for the development of EC. The mismatch genes, *MutL* Homolog 1 (*MLH1*), *MutS* Homolog 2 (*MSH2*), *MutS* Homolog 6 (*MSH6*) and *PMS1* Homolog 2, Mismatch Repair System Component (*PMS2*) are all component parts of the post-replicative DNA mismatch repair system (MMR) that coordinately repairs double stranded DNA errors. Mutations of these genes prevents DNA repair. * A hamartoma is a tumour-like, non-neoplastic disordered proliferation of mature tissue.

There are also hereditary syndromes that affect the gastrointestinal tract that predispose some women to simultaneous or later development of EC (Figure 5). Many of the genetic factors that induce EC are also responsible for these hereditary syndromes (Figure 5). Although the causes of these syndromes is still being undertaken, it is important to note here that the *POLD1* mutation is now known to be associated with increased risk of EC but the *POLE* mutation seems to be protective [63,70].

## 6. How Metformin and Progestin Protects against EC

Prominent mechanisms by which metformin combats EC appear to be through promotion of progesterone receptor expression and the reversal of progestin resistance [166,167]. Since EC is largely an oestrogen-driven disease, one of the treatments used is to administer progesterone or synthetic progestins, which counter the action of oestrogen in the endometrium. However, a major hurdle for this treatment approach is that the expression of the target for progesterone and synthetic progestins, the progesterone receptor, is sometimes downregulated in EC cells, especially following long-term treatment with a synthetic progestin or in patients with Type 2 EC. This negates the effects of progesterone or synthetic progestins, even if ample concentrations are available [168]. The simultaneous administration of metformin and the synthetic progestin medroxyprogesterone acetate (MPA) decrease proliferation of EC cells in culture [166], whereby metformin increased progesterone receptor expression allowing a place for MPA to bind. The MPA presumably inhibited oestrogen receptor alpha expression and so prevented oestrogen-dependent cell proliferation [169] although this was not studied. Similarly, Zhang and colleagues in 2011 demonstrated that metformin not only reversed progestin resistance in their model system but inhibited cell proliferation and induced apoptosis (programmed cell death) in progestin-resistant EC cells [167].

Natural progesterone also exerts several anti-cancer effects in endometrial tissue, primarily related to cell differentiation. In one experimental study, administration of progesterone to EC cells reduced cancer cell proliferation by activating the metabolic regulators p21 and p27 [170]. In addition, treatment with progesterone led to a reduction in the expression of several cellular adhesion molecules that cancer cells use to attach to normal tissues and spread [170]. In one study that followed 12 women with stage 1, grade 1, type 1 EC for up to 36 months, placement of a progesterone-containing intrauterine device resulted in negative biopsies at 12 months in 6 of 8 patients [171]. Progesterone also reduces the expression of the alpha form of the oestrogen receptor, which is major proliferation stimulator in EC cells [172]. Indeed, when the receptor is missing, activation of the beta form of the oestrogen receptor appears to inhibit EC cell proliferation [173].

Progesterone also augmented the anti-tumour effects of vitamin D by upregulating the expression of the vitamin D receptor in EC cells [174], whilst simultaneous administration of a metabolically active form of vitamin D (1,25-dihydroxyvitamin D3) and progesterone led to a significant upregulation of proteins that help restrain tumour growth and metastasis in EC cells [175]. These results suggest that women undergoing progesterone therapy for EC may be able to achieve a more desirable outcome by ensuring their blood levels of 25-hydroxyvitamin D are in the optimal range. Although studies have yet to test this hypothesis thoroughly, one effect may be to induce apoptosis through the reduction of reactive oxidative species [176]. Nevertheless, it is known that women in hotter, sunnier regions of the world have lower incidence of EC when compared to those from cooler, less sunny environments [40,41], suggesting this observation may be correct (the sun converts ingested vitamin D into the metabolically active form [177]). Additionally, complete covering of the skin for cultural or religious reasons can also reduce vitamin D levels and increase EC risk, suggesting a causal link [178].

## 7. Dietary Factors

EC appears to be especially influenced by dietary and lifestyle factors [24] with a variety of factors related to diet and lifestyle increasing the chances of developing EC; chief among them is the consumption of foods high in animal fats and sugars, whereas diets high in vegetables and fruits (especially those high in lutein) have lower risk [31,34,143,179,180]. High intake of iron from red meat has also been modestly associated with increased risk [181,182,183]. Since dietary factors are implicated in the development of EC, it is important to examine some of these factors in turn, starting with lipids.

### Lipids

Dietary omega fatty acid composition increases the risk of developing several diseases, including cardiovascular disease and cancer. There are two primary omega fatty acids: omega-3 lipids and omega-6 lipids, differentiated by their chemical structure. Omega-3 lipids are generally viewed as exerting anti-inflammatory action, whereas their omega-6 counterparts are easily metabolised into pro-inflammatory end products [184]. Given that inflammation plays a major role in tumour initiation and survival, omega-3 fatty acids have gained considerable attention in the context of cancer prevention and treatment [185]. Indeed, evidence suggests a higher dietary omega-3 to omega-6 ratio is associated with a lower risk of EC [186]. For example, in a study of 556 women with EC and 533 healthy controls whose consumption of the omega-3 lipids eicosapentaenoic acid (EPA) and docosahexaenoic acid (DHA) recorded a significantly lower EC risk. These are often found at high concentrations in cold water fish, and the women in the top 25% who ate fish rich in EPA had a 43% lower risk of developing EC, when compared with the women in the lowest 25%. Similarly, those consuming the most DHA had a 36% lower risk compared to those consuming the least, whilst a ratio of omega-3 to omega-6 fatty acids in a diet was associated with reduced EC risk. Finally, those women who consumed fish oil supplements had a 37% lower risk of EC [186]. A larger study of more than 3500 women found that eating fatty fish (which also contain high concentrations of omega-3 fatty acids) had a 40% lower EC risk [183]. These omega-3 fatty acids are thought to prevent cancer development by altering gene expression, oestrogen metabolism, and by causing improved insulin sensitivity and reduced inflammation [186,187]. This suggests that consumption of oily fish or supplementation of diets with a high omega-3 to omega-6 ratio would be beneficial. The source of that omega-3 may be of some concern since some sources may be contaminated with toxic/carcinogenic compounds that adversely affect the development and progression of other cancer types [188].

One of the healthiest eating styles in the world is the so-called “Mediterranean diet”, a traditional dietary pattern of populations of the Mediterranean region that includes the Southern European states, the North African states and those of the western fringes of the Middle East [189]. This diet centres upon whole grains, vegetables, fruits, olive oil, fish, and is combined with moderate dairy and wine consumption. The Mediterranean diet reduces the risk of several of today’s most prominent ailments including obesity, cardiovascular disease, and cancer [190,191]. Accordingly, rates of EC in the Mediterranean region are lower than in the United States and United Kingdom [40,41], and other ‘Westernised’ societies (Figure 2), and this effect is thought to be caused, at least in part, to differences in diet within these regions. In fact, it has been estimated that 10% of EC cases could be prevented if “Western” societies shifted to a more Mediterranean-like diet, one with less red meat and animal fat [192]. In one study, adherence to a “Western” diet, which is high in saturated and animal fats (as well as refined carbohydrates), was associated with a 60% increased risk of developing EC [193], whilst another study of Italian women consuming a Mediterranean diet with low adherence showed an approximate doubling in EC risk compared to a control cohort that had high adherence rates, with an increased dietary inflammation index (the consumption of foods with inflammatory properties such as red meat and processed meats) being the intermediary cause [194]. Indeed, a meta-analysis of available data from case-control studies on the risk of developing EC due to the consumption of red meat concluded that higher red meat consumption of 100 g per day increased EC risk by 84% [195]. By contrast, the same authors reported there to be no association in prospective observational studies, suggesting that an association of EC with red meat consumption is probable rather than absolute. Indeed, in a meta-analysis of multiple studies, a 51% increased EC risk was associated with red meat consumption but there was no association with white meat (chicken) or fish consumption [196] supporting the idea that a Mediterranean-like diet would be beneficial. Other cohort studies, e.g., the Iowa Women’s Health Study indicated no association between red meat consumption and EC risk [197]. This provides inconclusive evidence for a causative role of red meat consumption in the risk of developing EC. However, it should be pointed out that analyses from this cohort also failed to find an association between red meat and colorectal cancer, which has generally been supported by other cohort studies to be an important association [198,199]. All of these disparate studies have provided a less than ideal understanding on whether red meat or processed meat consumption is a ‘true’ risk factor for the development of EC and that a vegan or vegetable/vegetarian-only diet may be preventative of EC development. Current knowledge, however, does not support that hypothesis [200]. The EPIC-Oxford trial, which ran from 1993 to 2005 demonstrated that the incidences of ovarian, uterine, endometrial and breast cancers were not significantly different to their omnivore counterparts, even when fish was allowed to be part of the vegetarian diet [201,202]. Additionally, the Adventist Health Study of Californian vegan/vegetarians that ran from 1976 to 1982 indicated a modest 15% reduction in EC risk, although the risk difference was not significant (OR 95% CI range was 0.58 to 1.23) [203]. The available data so far suggests that a more vegetable-fruit based diet may be more protective against the development of EC, but data supporting a recommendation along these lines for EC protection remains elusive. To reduce red meat intake and to eat more fruit and vegetables (in moderation) is supported, since doing so prevents other forms of cancer and prevents other life-threatening diseases [203,204,205,206,207,208], presumably because doing so reduces an individual’s body mass and changes body composition whilst providing essential vitamins and minerals that cannot be obtained from meat products [209,210].

## 8. Vitamins and Minerals

### 8.1. Vitamin A and Carotenoids

Vitamin A is an essential vitamin that is found in plants and stored in the liver. It is part of a family of pigments found in plants called the carotenoids, which are normally yellow. The most well know of the carotenoids is called beta-carotene, which under the actions of bile acids is converted in retinal, then into the active form of vitamin A. Vitamin A and some vitamin A derivatives, are active at retinoic acid receptors where they act as transcriptional regulators [211]. On activation, retinoic acid receptors alter the transcription of many genes involved in inhibiting tumour growth and invasion, through inhibition of carcinogenesis and induction of programmed cell death (apoptosis) [212,213]. Increased ingestion of vitamin A or β-carotene is associated with a lower risk of developing EC [143,179,214,215]. When consumption of beta-carotene is combined with vitamin C, the risk of developing EC is halved [216]. It is for this reason that all cancer patients are often advised to increase their consumption of vegetables that are yellow and orange [215], even though conclusive evidence is still lacking [217]. Preclinical studies of some carotenoids have demonstrated that they can synergistically affect the therapeutic potential of some anti-cancer drugs. For example, the carotenoid, fucoxanthin enhances the cytotoxicity of doxorubicin in multi-drug resistant breast cancer cells [218], suggesting that certain vitamin-therapeutic combinations could be more effective in preventing some forms of neoplastic recurrence. A corollary to that idea is that some carotenoid treatments may affect other tissues, such as bone whilst also protecting against breast and EC neoplasia [219]. More research into the effects of vitamin A and the carotenoids would therefore be useful.

### 8.2. Vitamin C

As stated above, vitamin C may aid in the prevention of EC. This vitamin (ascorbic acid) has been reported in many studies to significantly reduce the risk of developing EC [28,32,214,220,221,222]. Vitamin C has been proposed to reduce the activity of a key protein called hypoxia inducible factor-1 alpha (HIF-1α), which is involved in endometrial tumour cell survival [221,223]. Although vitamin C has direct effects on tumour cells, it also may have an effect on tumour biology by modulating the actions of the immune system, in which tumour cell surveillance is improved and tumour cell killing is enhanced [224]. Increased vitamin C consumption not only reduces EC incidence, but prevents the advancement to more aggressive disease grades [214,220,221]. In 2009, Bandera and colleagues demonstrated that only 50 mg vitamin C per 1000 calories consumed reduced EC risk by 15% [220]. A higher level of vitamin C (≥72.7 mg of vitamin C per 1000 calories/day) reduced EC risk by 20%. Women in the lowest consumption group (≤29.8 mg per 1000 calories/day) had the highest EC risk [214]. These data suggest that foods rich in vitamin C, such as cauliflower, kale, pineapple, sweet potato, bell peppers, peas, legumes, strawberries and citrus fruits should be part of the daily diet [4,225]. In a meta-analysis of numerous observational studies, vitamin C consumption was able to reduce breast cancer incidence by 11% when part of the daily diet, but a similar effect with vitamin C supplements was not [226]. More importantly, the study also showed that breast cancer recurrence remained lower in the higher vitamin C consumption group when compared with the lowest consumption group [226]. These observations have been confirmed in several other recent studies where vitamin C appears to enhance both radio- and immuno-therapy regimens, possibly through alterations in the neutrophil-macrophage ratio [227,228,229,230]. Similar data for patients with EC are lacking, but the recent demonstration of a similar effect of vitamin D (an effector for the immune system) on EC outcomes [231,232], suggest that vitamin C or D consumption may be beneficial for many forms of cancer [233] including EC. Indeed, this idea was proposed many years ago [234].

### 8.3. Vitamin E

It is now clear that in vitamin E consumption results in a significantly reduced risk of developing EC [214,215]. Many foods contain significant amounts of vitamin E. Wheat germ and other seed bearing oils contain the highest amounts of vitamin E. Nuts such as almonds, hazelnuts, pine nuts and sunflower seeds, which are moderately high in vitamin E, and vegetables such as sweet red peppers, tomatoes and spinach contain lower, but significant levels of vitamin E, but each should be part of the vitamin diet. High intakes of vitamin E has been reported to cause a 56% reduction in EC risk [215]. Despite these data, larger prospective cohort studies of 87,998 women in the Nurses’ Health Study and 47,344 men in the Health Professionals Follow-up Study failed to replicate these results [235]. Although some research links higher intakes of vitamin E with decreased incidence of breast cancer, an examination of the impact of dietary factors, including vitamin E, on the incidence of postmenopausal breast cancer in >18,000 women found no benefit from the vitamin [236].

Vitamin E naturally occurs in more than one form, with each form having a different level of biological activity) [237]. Of the eight chemical forms (alpha-, beta-, gamma-, and delta-tocopherol and alpha-, beta-, gamma-, and delta-tocotrienol, only gamma-tocopherol had a significant anti-inflammatory and anti-tumour effect in a rat model of breast cancer [238]. This is of interest when considering EC development and risk because gamma-tocopherol appeared to work through inhibiting oestrogen activity in other gynaecological cancers and so inhibits tumour growth. For example, Korean women who consumed higher amounts of gamma-tocopherol showed a 72% lower risk of developing ovarian cancer compared to women who have not [239]. Although the effect of vitamin E and other anti-oxidant vitamins on the prevention of DNA damage is modest [240], they do have a contributory role in cancer prevention [241,242,243] that is starting to be exploited therapeutically [244].

### 8.4. Selenium

Selenium is an essential micronutrient required for numerous metabolic processes throughout the body [245]. Gynaecological cancers, such as those of the uterus and cervix are less prevalent in women exposed to higher levels of selenium than those who have low level exposure [246,247]. In 2009, a randomised prospective clinical trial showed sodium selenite supplementation to be advantageous for cervical and uterine cancer patients who had selenium deficiency, because it reduced the diarrhoea caused by radiotherapy treatment [248]. Selenium supplementation may be particularly useful for cervical cancer patients, because in vitro studies have shown that sodium selenite induces cervical cancer cell apoptosis [249]. Other studies have shown that selenium can disrupt oestrogen signalling in cancer cells [250]. Furthermore, additional more recent in vitro studies have revealed that selenium can also act together with vitamin C to prevent tumour cell growth in both breast and colon carcinoma cell lines [241,251,252]. Nevertheless, more studies in this area are needed and assessment of the daily therapeutic level for the prevention of EC is required.

### 8.5. Calcium

Everybody knows that calcium is good for healthy teeth and bone. It is found in many dairy products and in its ionic form is intimately involved in the release of hormones from endocrine glands, neurotransmitters from nerves, initiating muscle contraction, and for maintaining bone [253]. It is also a critical component of protein kinase C (PKC) signalling, which controls many cellular growth and apoptosis pathways [254]. Cellular differentiation and proliferation must be carefully regulated otherwise cancers develop and calcium plays a critical role in controlling many of the metabolic pathways here [255]. The risk of developing EC is greatly reduced in women taking calcium supplements or who consume calcium-rich foods. Dairy foods such as milk, cheese and yoghurt, and non-dairy products such as seafood, leafy greens, legumes, dried fruit and tofu have all been shown to significantly reduce EC risk [36,183,256], suggesting that research into calcium supplementation in the prevention and potential treatment of EC would be useful as it is for Korean breast cancer survivors who show longer survival when supplements containing calcium are consumed [257].

### 8.6. Cadmium

Cadmium is a chemical element that is ubiquitous in Nature and is found in air, water, soil and plants. It is also extracted from rocks and processed in order to make the expanding nickel-cadmium (Ni-Cd) battery industry. These batteries are increasingly used in many modern electronic devices. Exposure to cadmium is greater in industries where it is processed and ingested from contaminated hands, from plants that absorb it from the environment, or from cigarette smoking [258]. In the context of cancer, cadmium exposure has been associated with lung, kidney and prostate cancer [259,260]. It is also been associated with decreased EC risk due to the actions of the Cd^2+^ ion which acts a weak oestrogen [261,262], binding to and preventing the actions of oestrogen receptor alpha [263]. Studies have indicated that smoking per se protects against EC [264,265] in both prospective and case-control studies, which showed the greatest decrease of risk occurred in heavy smokers over increasing years of use [266]. By contrast, other oestrogen-specific cancers, such as ER-positive breast cancer is not protected nor exacerbated by Cd^2+^ ions [267] despite in vitro studies that indicate it may increase breast cancer cell malignancy through a decreased dependency on oestrogen-stimulated cell proliferation [268]. Despite this potentially beneficial effect of cigarette smoking for women at risk of developing EC, the other detrimental health effects (cardiovascular disease, tumour formation in the respiratory tract and lungs, etc.) outweigh this potential benefit.

In conclusion, the role of vitamins and minerals in providing protection against EC is only partially supported by scientific consensus [36,37] and although the evidence is scant, more detailed studies in this interesting area should help to clarify the role(s), if any, of these essential micronutrients in the near future [200].

## 9. Plant Derivatives and Hormones

### 9.1. Lignans

Phytoestrogens have long been known to ameliorate perimenopausal symptoms and have significant health benefits in postmenopausal women. The key natural phytoestrogens found in plants such as flaxseed and sesame are the lignans [269]. The active ingredient in such foods are not the lignans themselves, but their metabolised product, enterolactone. This compound has two important effects in cancers, (1) it promotes cancer cell death and (2) prevents the growth of new blood vessels in hormone-responsive tumours thereby prevents the tumour’s capacity to allow cancer cells to grow [270]. It has been postulated that phytoestrogens may compete with endogenous oestrogen for binding at the oestrogen receptor [271,272], which is logical since the most common form of EC is type 1 EC, which is oestrogen-dependent. Thus, studies showing high lignin consumption in all types of women have a 32% lower risk of developing EC, support this hypothesis. It is further supported by a study in postmenopausal women, where EC risk was 43% lower [273]. It has been historically impossible to recommend phytoestrogen consumption to postmenopausal women, since such consumption might also induce breast cancer in oestrogen receptor positive neoplasms, however, several studies have shown this idea to be unfounded and that the phytoestrogen enterolactone is protective against breast cancer too [274,275,276].

### 9.2. Soy Isoflavones

Soya beans and other legumes contain significant amounts of isoflavones, as do green tea, red clover, lentils and flaxseeds. Increased ingestion of such food results in lower EC risk [277]. Postmenopausal women were significantly less likely to develop EC if they consumed more soy isoflavones (including genistein and daidzein) and total isoflavones [277]. Additionally, a lower risk of developing EC has been reported in several case-control studies where soy and legume consumption was high [180,278,279]. Soy isoflavones bind to oestrogen receptors and modulate intracellular oestrogen signalling, whereby they are similar to lignans and compete with endogenous oestrogens, to reduce endogenous oestrogenic activity [272,280], however, three recent studies have shown that soy ingestion may increase the risk of developing breast cancer [21,281,282], whilst ovarian cancer risk was reduced [283]. So far, available evidence does not point to any dangers from consuming soy, with general health benefits appearing to outweigh any potential risk [284]. In fact, there is growing evidence that eating traditional soy foods such as tofu, tempeh, edamame, miso, and soymilk from an early age may lower the risk of developing EC and breast cancer, especially among Asian women [285,286]. Soy foods are excellent sources of protein, especially when they replace other, less healthy sources, such as animal fats and red or processed meats. The reason why some studies have shown an increased risk of developing breast cancer through ingestion of isoflavones [281,287], whilst other studies have not confirmed such associations or have shown the opposite [179,180,277,279,283,285,286,288,289], is probably due to the presence of the oestrogen receptors in the starting neoplasm [281], the population under study [290] or the physical form that the isoflavone that is consumed [288,291]. Meta-analyses of different studies in various populations generally support the idea that moderate to high consumption of a diet that contains isoflavones prevents EC, ovarian and breast cancer and its recurrence [283,292,293,294]. These data suggest that a diet rich in isoflavones or that concentrated soy supplements, which contain much higher isoflavone concentrations will be beneficial, but until more research is done with pre- and peri- and postmenopausal cohorts, a definitive conclusion cannot be met.

### 9.3. Coffee and Chlorogenic Acid

Coffee consumption has been linked with a reduction in the development of all types of oestrogen-driven cancers, including EC [295,296,297,298,299,300,301], where drinking at least four cups of coffee per day causes a 25% reduction EC risk when compared to drinking one cup or less per day. Interestingly, because drinking equivalent amounts of decaffeinated coffee per day (two or more cups) was associated with a 22% reduction in EC risk [302], then the effect is not due to the presence of caffeine or its primary metabolites, but due to other constituents of the coffee bean. When the bean is ground and filtered, EC rates dropped, if the ground bean is boiled then EC rates increased [303]. These data suggest that modest coffee consumption could be beneficial in the prevention of EC, however, what effect, if any coffee consumption has on progression or disease recurrence is currently unknown. Conversely, drinking brewed or instant coffee has been associated with an increased risk of developing breast cancer in some [304,305] but not all studies [306] and has no effect on the incidence of ovarian cancer [307]. In these studies, it appears that exposure of the coffee bean or its processed products to heat is a preventative factor, since instant and boiled coffee showed a 50% reduction in the risk of developing breast cancer, whilst filtered coffee seems to have the opposite effect, especially in premenopausal women of normal weight [303,305]. Paradoxically filtered coffee has the opposite effect in postmenopausal women, where there was a 40% reduced risk of developing breast cancer, but an increased risk if the coffee was instant or boiled [303], suggesting that patients at risk of developing endometrial cancer (who are generally postmenopausal) should drink moderate amounts (no more than three cups) of filtered coffee per day [301]. Although coffee contains multiple phytochemicals and polyphenols that exert various physiological effects, the polyphenol chlorogenic acid (CGA), is the probable active ingredient in respect to EC protection since it may protect cells from oxidative DNA damage [308]. Although CGA concentrations are moderate in brewed coffee, it is highly concentrated in green coffee bean extracts, explaining the protective effect of filtered coffee over brewed or instant coffee. The coffee bean and its extracts may also have indirect effects in preventing EC, since it inhibits insulin production, improves insulin resistance [309] and so is beneficial to EC patients because it prevents weight gain and modulates glucose metabolism [30,302,310]. Obviously, such observations need corroboration in larger studies and all forms of cancer need to be considered, especially as boiled coffee is associated with respiratory tract cancers in men [303]. That this final effect is confined to men, suggests a gender-specific factor is interacting with a component in coffee to induce or exacerbate the neoplasms found in men, whilst a different gender-specific factor is protective in women.

### 9.4. Green Tea and (−)-Epigallocatechin-3-Gallate

Drinking green tea has historically been associated with reduced incidence of many cancers [311,312]. The major polyphenol found in green tea, epigallocatechin-3-gallate (EGCG), was reported by Kakuta and colleagues to be effective against EC, and that effect was independent of other EC risk factors such as obesity or menopause [313]. In preclinical studies, EGCG inhibits EC cell proliferation and induces cell death [314] and in a single animal study, EGCG inhibited angiogenesis [315], thereby preventing the formation of new blood vessels that support large tumours. Interest in this has expanded and a meta-analysis of 7 published studies reported that the protective effect of green tea was better than that of black tea and that a 25% decrease in EC risk could be achieved by drinking an additional 2 cups of green tea per day [316] (Table 4). More recent evidence using a xenograft model indicated that the use of a prodrug form of EGCG inhibited tumour cell proliferation and increased apoptosis in a time- and dose-dependent manner [317]. One effect was that microvessel formation was decreased, thereby reducing blood flow to the xenograft. Similar effects were observed in a mouse model of ovarian cancer [318], suggesting that careful use of such extracts could be useful for the prevention of numerous neoplasms.

### 9.5. Agaricus Mushroom

The agaricus mushroom (*Agaricus blazei Murill Kyowa*, ABMK) looks a little like a chestnut mushroom and the family includes the common button mushroom used in kitchens throughout the world. This particular mushroom and its extracts has been studied in cancer patients in two clinical trials. In one study of 100 women with gynecological cancers, including those with EC, found that chemotherapy could be improved by 6 months supplementation with ABMK extract whereby there was increased anti-cancer immune natural killer cell activity [338]. Furthermore, chemotherapy side effects such as emotional instability, hair loss and loss of appetite were drastically reduced [338]. In 2011, Ohno and colleagues examined the effect of ABMK extract on 78 cancer patients in remission and found that a diet supplemented with 1.8–5.4 g per day also benefitted from exposure to the extract, but importantly the extract was well tolerated (in most subjects), indicating that this product is generally safe [339]. The beneficial effect of these fungal extracts is not confined to EC, since recent evidence indicates they are efficacious and well-tolerated in breast cancer [340,341] and prostate cancer patients [230], An in vitro study on three different EC cell lines indicated that extracts from three different mushrooms including the agaricus mushroom, had an inhibitory effect on cell viability and proliferation most probably through the induction of autophagy [342]. Further studies on the efficacy of agaricus extract supplementation in EC patients, both before and after diagnosis is therefore warranted.

### 9.6. Resveratrol

Resveratrol is a polyphenol molecule first identified n Japanese knotweed (*Polygonum cuspidatum*) but is now more commonly extracted from the skins of grapes. In vitro studies using several uterine models indicate that resveratrol not only inhibits cancer growth, but also initiates apoptosis [343]. In endometrial adenocarcinoma cell lines, the effects of resveratrol on cell growth and apoptosis appear to be both oestrogen-dependent and oestrogen-independent [344]. In addition, animal studies revealed that resveratrol and EGCG significantly reduced VEGF secretion by EC cells in a concentration-dependent manner, indicating resveratrol and EGCG could provide effective anti-angiogenesis therapy in patients with EC [345]. Resveratrol is found in wine, particularly red wine and is thought to also act as an antagonist at the oestrogen receptor alpha [346], thereby blocking the actions of exogenous and endogenous oestrogens. It is also an agonist at the oestrogen receptor beta, which is known to be an inhibitor of EC cell proliferation and inducer of apoptosis is other oestrogen-sensitive tissues [347]. Data from epidemiological studies indicates that consumption of three or more drinks of alcohol per day increases the risk of developing breast cancer regardless of type [348], yet some caution is advised because in vitro studies of EC cells indicated that the beneficial effect of resveratrol could be prevented if the cells undergo autophagy [349] prompting the authors to recommend that chloroquine (an autophagy inhibitor) be used to prevent the autophagy during treatment since the combination enhanced growth inhibition and cellular apoptosis.

### 9.7. Curcumin

Curcumin is chemical constituent of the spice turmeric that is used widely in South and Southeast Asian cuisine. It is also a food additive for yellow-orange coloured food in EU (E number 100) that significantly inhibits cancer cell proliferation through numerous cellular targets [350]. It also has the ability to improve insulin metabolism, and may prevent obesity-related cancers, such as EC [351]. Curcumin acts by preventing the phosphorylation of a protein (STAT-3) that acts as a transcriptional regulator intimately associated with the uncontrolled growth of cancer cells [352]. Moreover, curcumin’s ability to induce apoptosis of human EC cells by the dysregulation of proto-oncogenes [353], is being exploited in a clinical trial that is due to complete in 2022 [354]. Because curcumin induces apoptosis and prevents cell cycle progression at the G2/M phase of the cell cycle in oestrogen receptor positive breast cancer cells [355] and triple negative breast cancer cells [356], it is likely a similar effect occurs in endometrial cancer cells too [357].

### 9.8. Indole-3-Carbinol and Di-Indoylmethane

Cruciferous vegetables such as cabbage, cauliflower, radishes, broccoli and Brussel sprouts all contain high concentrations of indole-3-carbinol (I3C), which in vivo is rapidly converted into diindoylmethane (DIM) [358]. DIM possesses anti-cancer properties in breast, endometrium and prostate cancers [358,359]. Because both I3C and DIM can interfere with oestrogen metabolism and signalling, they are proposed to be good candidate drugs for the treatment of hormonally-responsive cancers, and especially EC. These two compounds prevent the conversion of oestrogens into 16-hydroxyoestrogens, but promote their conversion into 2-hydroxyoestrogens. These results in two effects: (1) the stronger promotion of cellular proliferation by 16-hydroxyoestrogens is blunted, and (2) hormone-responsive cell growth is reduced because 2-hydroxyoestrogens are weaker activators of the oestrogen receptor, and consequently the cells undergo less proliferation [359,360,361,362,363,364]. I3C and/or its metabolites also modulate some metabolic pathways that are critical to cancer cell survival [358] and DIM may interrupt oestrogen receptor signalling in EC cells [365]. In combination with the soy isoflavone genistein, programmed cell death is enhanced through increased TRAIL action, a protein that has cancer-cell-killing properties [366].

Rats genetically prone to developing EC, and given a diet for 660 days that was supplemented with I3C, had a lower EC rate when compared to similar rats fed a standard diet [251]. The authors showed that rats receiving the highest I3C dose, had a lower EC rate (14%) compared to that of the standard-diet group (38%). Ingested I3C significantly increased the 2-hydroxylation of oestradiol, thereby reducing circulating oestrogen concentrations [367]).

### 9.9. Melatonin

Melatonin, is a natural hormone produced by the pineal gland that regulates energy metabolism and sleep patterns [368]. It is produced naturally through darkness and through effective sleep patterns. Melatonin may also help prevent the development of tumours that are responsive to sex steroid hormones, such as those of the prostate, breast, and most gynaecological cancers (EC, cervical and ovarian cancer) [369,370] and breast cancer [371]. Patients with non-small cell lung cancer benefit from additional melatonin therapy because it also improves the effectiveness of chemotherapy [369,372,373,374,375]. Several studies have shown that melatonin has many anti-cancer activities, such as directly promoting cancer cell death and independently promoting immune responses against some tumour cells [376], but not all [377,378]. Melatonin release into the blood is highest in the early hours of the morning and as such, a lifestyle that includes regular sleep patterns is important because of its regulation of energy balance and metabolism [379]. It has three major effects with respect to EC: (1) it acts as a p450 aromatase inhibitor and so stops the production of oestrogen from androgens [380], (2) it destroys free radicals, which can cause DNA, lipid and protein damage [381,382] and (3) it enhances the anti-tumour activity of vitamin D [383], which we have already seen enhances the anti-EC effects of progestogens, by increasing the expression of the progesterone receptor in endometrial cells.

In conclusion, the role of plant derived materials and hormones in the incidence of EC is stronger than that for vitamins and minerals. Some organisations, such as the IARC [41], the World Cancer Research Fund [4] and the World Health Organization [40] support the notion that some plant derivatives, especially coffee and tea, and other substances high in anti-oxidative power are beneficial and prevent the development of EC.

## 10. Clinical Trials and Studies

It is been known for many years that a diet rich in mono-saturated and trans-fats are detrimental to the development of cancers and that diets rich in plant foods are beneficial [384], yet there are very few clinical trials that identify ‘good’ foods that benefit patients already diagnosed with EC, only those that indicate what should be avoided.

The biggest study designed to investigate the relationship between nutrition and cancer is the European Prospective Investigation into Cancer and Nutrition (EPIC) study, which is an ongoing multi-centre prospective cohort study [385]. It is well suited to study the influence of diet on cancer risk because of the large variation in dietary patterns across the 10 collaborating Western European countries [386]. Cust and colleagues in an interim report gathered from the EPIC study examined the effect of carbohydrate on the development of EC, where they concluded that there is no association of overall glycaemic index, total starch, or total fibre intake with the risk of developing EC [387], but that there is a possible modest positive association of total carbohydrates, total dietary glycaemic load, and total sugars with risk of developing EC, particularly amongst women who have never used hormone replacement therapy [387]. They also demonstrated that glucose, plasma lipid and lipoproteins in the context of metabolic syndrome were risk factors in the same cohort [97].

Aarestrup and colleagues in 2012 conducted a study investigating the association of whole grains and dietary fibre with the incidence of EC in Danish women and showed there to be no clear associations between intake of whole grains or dietary fibre and the incidence of EC [388]. In addition, a similar study conducted in Western New York by McCann and colleagues in 2000, discovered a reduced risk of developing EC was associated with a diet rich in plant food [222]. A case-control study in Mexico suggested dietary vitamin D and calcium might play an important role in reducing the development of EC but no association between the consumption of animal or vegetable proteins and fats with EC was found [256].

These studies performed in China, the United States of America, Canada, Mexico and Denmark provide limited and inconclusive evidence regarding the association of these modifiable factors with the risk of developing EC. To our knowledge, no study has yet been performed to determine the association of dietary pattern and lifestyle habit with the risk of developing EC among women in the UK, although there is an on-going study (ISRCTN1538157) that is examining the effect of general weight loss through a 12 month weight loss programme on the incidence of endometrial cancer (and breast cancer); the results are awaiting trial completion in 2023. In women previously diagnosed with endometrial cancer, a pilot trial (NCT02433080) on the general effect of diet and exercise on quality of life by questionnaire demonstrated a significantly improved quality of life (difference of 8.9) at 24 weeks, but suffered from small sample numbers (29 in the intervention group and 31 in the control group), with incomplete follow-up [389]. In the USA, a similar study of 196 obese endometrial cancer survivors demonstrated that a combination of weight loss and the use of technology in the form a text-based app provided sufficient motivation for a 4.4 kg weight loss that increased the patients’ quality of life [390], suggesting that encouragement of weight loss in endometrial cancer survivors, either by dietary changes [391], increased physical activity [392] or enhancement of usual care can be beneficial for such patients [390]. Such interventions in EC survivors may even prevent disease recurrence, although definitive conclusions or studies supporting such a conclusion are lacking. Other studies in EC survivors have either been withdrawn (NCT03042897), are on-going (NCT02465541; NCT01697566; NCT04534309) or have completed (NCT00420979; NCT00587886; NCT01610375; NCT04000880) without clear conclusions that exercise or dietary changes can improve a patient’s quality of life [393,394] or are designed to test existing interventions, such as the use of metformin as a chemo-preventative agent (NCT01697566; NCT02431676). Therefore, we suggest that further studies need to be designed to explore the association between the lifestyle habits and dietary intake among women with the risk of developing EC here in the UK, especially as regional differences in EC incidence, prevalence and mortality appear to be changing around the world.

In an on-going phase II clinical study (NCT03192059), curcumin is under evaluation as an adjuvant (food supplement) in patients with advanced and/or refractory cervical cancer, endometrial carcinoma or uterine sarcoma who are simultaneously being treated with an immunomodulatory cocktail (vitamin D, aspirin, cyclophosphamide and lansoprazole), followed by pembrolizumab, combined with radiation [354]. No preliminary data are available yet, but this cocktail is expected to yield positive results.

## 11. Current Research Models and Future Research Directions

In preparing this manuscript it has become increasingly obvious that research into the effects of nutrition, diet and lifestyle on the prevention of cancer and general health is difficult [395]. Although we have summarised here the relatively few better-established links between nutrition, lifestyle changes and the prevention of several cancers, what the most important risk factors with respect to EC appear to be obesity and a sedentary lifestyle [35,90]. Which diets or lifestyle corrections can be made or which specific food components or broader dietary patterns, such as the so called plant-based diets, will be important in the future remains unclear [37,180,193].

To move forward, a new generation of studies need to be created to improve estimates of long-term exposure with, for example, repeated dietary records, which are now feasible using web-based questionnaires [396,397] even though these are rarely used in place of the food frequency questionnaires that suffer from recall bias [398,399]. Cancer-specific biomarkers of dietary intake and nutritional status should be used more extensively, and new cancer tissue biomarkers might be found through proteomics and metabolomics studies, for example, but these too are problematic in that anything found will still need to be validated and interpreted in the light of possible confounding and reverse causations. For some exposures, both for intake and nutritional status, Mendelian randomisation has been suggested to be able to clarify causality [400]. Of course, when something has been identified to be linked to the reduction of EC risk or improves prognosis, randomised trials will be needed to test specific hypotheses. It will also be important to attempt to coordinate systematic analyses of all the data available worldwide, in order to reduce the risk of population bias [401]. For public health and healthcare policy makers, the top priority should be tackling the known major diet-related risk factors for EC, particularly obesity and alcohol [200].

In all of these cases, the best model for study is the patient at highest risk of developing EC and that is the adult woman. Although other models, such as human and animal cell lines, rodent, primate and other animal models (including xenobiotic implants) are useful in defining the molecular mechanisms and physiology of interactions between food, nutrients and EC risk, they are not as useful for studying interactions between lifestyle interventions (such as regular exercise, weight loss programs or getting a good night’s sleep), even if the recovering EC patient does not adhere to advice [402]. There is still a long way to go.

## 12. Conclusions

Although there is substantial evidence that moderate levels of physical activity, bearing children, delayed menopause and eating foods that are rich in lutein (fruits and vegetables) can protect against obesity and the associated conditions of hyperinsulinaemia and metabolic syndrome and the risk of developing type 2 diabetes mellitus [323] and that alcohol consumption may increase, and coffee and tea consumption may reduce, the risk of EC [307,326,403], these interventions have not yet filtered through to the patients at risk. More evidence is thus required for meta-analysis of the individual factors that prevent EC incidence [4] and that will come from further study. A key conclusion therefore is that although some clinical trials support dietary and lifestyle alterations, there is room for more to be conducted.

Recommendations for women wishing to prevent EC and for those wishing to prevent recurrence, are difficult and although the global incidence of EC is increasing, prevention can be achieved if the patient modifies her diet and lifestyle. Women at risk should; (1) lose weight (if overweight or obese (as many EC patients are)) since doing so can reverse their risk of developing hyperinsulinaemia; (2) eat a diet rich in protein but with a low GI that contains wholegrains, vegetables, fruits and legumes, whilst reducing their consumption of foods that are high in fat, sugar or starch, red or processed meats. They should also (3) restrict their alcohol consumption, but drink 2–3 cups of filter coffee per day instead, because this aids the ‘slimming’ process and may reduce the risk of recurrent disease, or drink 1–2 cups of green tea, and although the evidence is limited, (4) make sure that she spends at least 20 min every day doing some form of moderate to intense exercise, since this aids the ‘slimming process’ and reduces the risk of entering a chronic inflammatory state (a clear risk factor for all forms of cancer), but especially gynaecological cancers such as EC.

## Figures and Tables

**Table 1:** The effect of income levels on the worldwide incidence, prevalence and mortality values for endometrial cancer in 2018.

Population	Incidence			Prevalence		Mortality		
	Number	** Crude Rate	** Age-Standardised Rate	5-Year	** Proportions	Number	** Crude Rate	** Age-Standardised Rate
High Income	173,957	28.5	15.5	670,689	109.8	36,911	6.0	2.4
Upper Middle Income	146,537	11.3	8.2	451,728	34.7	31,725	2.4	1.6
Lower Middle Income	52,535	3.6	3.9	140,955	9.5	17,632	1.2	1.3
Low Income	7,169	1.9	3.2	13,968	3.7	3,331	0.88	1.6

**—per 100,000; Data were taken from [40].

**Table 2:** Pathological, morphological, endocrine and common molecular genetic changes used to provide a dualistic clinical classification of endometrial cancers (Bokhman Classification).

Factor	Type 1 EC	Type 2 EC	References
Frequency distribution	80–90%	10–20%	[71,74]
Prognosis	Favourable	Unfavourable	
Outcome (5-year survival)	86%	59%	
Onset of menopause	≥50 years of age	≤50 years of age	
Obesity, hyperlipidaemia and diabetes mellitus association	Yes	No	
Association with oestrogen stimulation	Yes	No	
Sensitivity to progestagens	High	Low	
Background endometrium histology	Hyperplasia	Atrophy	
Tumour grade	Low (grade 1–2)	High (grade 3)	
Myometrial invasion	Superficial	Deep	
Potential for metastatic spread through lymphatic system	Low	High	
Reproductive status	Decreased	No disturbance	
**Clinicopathologic and molecular alterations**			
Prototypical histological type	Endometrioid	Non-endometrioid (serous, clear cell carcinoma)	[71,75,76,77,78]
Stage at diagnosis	Early (FIGO stage I–II)	Advanced (FIGO stage III–IV)	
ER and PR receptor expression	High	Low	
**Common genetic alterations**			
Microsatellite instability *	28–40%	0–2%	[66,70,71,79,80,81,82,83]
*PTEN* mutation	52–78%	1–11%	
*TP53* mutation	9–12%	60–91%	
*POLE* mutation	11–20%	0–7%	
*KRAS* mutation	15–43%	2–8%	
*PIK3CA* mutation	36–52%	24–42%	
*PIK3R1* mutation	21–43%	0–12%	
*ARID1A* mutation	25–48%	6–11%	
*CTNNB1* mutation	23–24%	0–3%	
*PPP2R1A* mutation	5–7%	15–43%	
*ERRB2* mutation	1–4%	26–44%	
*HER2* amplification	0	27–44%	

* Microsatellite instability is characterised by a deficiency in DNA repair mechanisms.

**Table 4:** Risk factors tested in patients with endometrial cancer and analysed through meta-analysis or Cochrane review.

Factor	Ref	Studies Included	Odds Ratio or Relative Risk (95% CI)	Direction of Risk
Alcohol ^a^	[319] [320]	14 case-control 7 cohort design	0.89 (0.76–1.05) 1.17 (0.93–1.46)	No effect No effect
Aspirin ^b^	[321]	5 case-control 4 cohort design Overall	0.82 (0.71–0.96) 0.91 (0.80–1.03) 0.87 (0.79–0.96)	↓ No effect ↓
Beast feeding	[322]	4 prospective and 10 retrospective studies	0.77 (0.62–0.96)	↓
Body mass index ^c^	[323]	Randomised clinical trial	RR 19.03 (1.17–310.52)	↑
Cadmium ^d^	[324]	4 cohort design	1.40 (1.06–1.84)	↑
Calcium	[255]	3 case-control	0.62 (0.42–0.93)	↓
Cholesterol	[109]	9 case-control 1 cohort design	1.07 (1.01–1.13) 1.06 (1.00–1.12)	↑ ↑
Cigarette Smoking	[325]	24 case-control 10 prospective	0.72 (0.66–0.79) 0.81 (0.74–0.88)	↓ ↓
Coffee (caffeinated vs. de-caffeinated)	[326]	10 case-control 4 cohort design	0.79 (0.73, 0.87) 0.65 (0.50, 0.85) ^e^ 0.76 (0.62, 0.93) ^f^	↓ ↓ ↓
Dairy products	[196]	8 case-control	0.97 (0.93–1.01)	No effect
Green tea	[316] [327]	7 case-control 6 cohort design	0.78 (0.66–0.92) 0.97 (0.82–1.14)	↓ No effect
Insulin resistance ^g^	[328]	2 case-control 2 prospective 21 cross-sectional	37.52 pmol/L (16.64–58.40) 33.94 pmol/L (15.04–52.85) 21.25 pmol/L (1.45–41.05)	↑ ↑ ↑
Lignans	[329]	3 case-control	0.92 (0.71, 1.20)	No effect
Lipids	[330]	14 case-control 7 cohort	1.21 (1.01–1.45) 0.91 (0.83–1.01)	↑ No effect
Metabolic syndrome	[331]	3 case-control 1 nested case-control 1 prospective 1 cohort registry Overall	2.45 (1.81–3.08) 1.62 (1.08–2.42) 1.37 (1.28–1.46) 2.77 (1.74–4.40) 1.89 (1.34–2.67)	↑ ↑ ↑ ↑ ↑
Oestrogen only HRT	[27] [332]	1 case-control 30 cohort design	1.45 [1.02–2.06] 2.30 (2.10–2.50)	↑ ↑
Oestrogen plus progestagens HRT	[332]	30 cohort design	0.40 (0.20–0.60)	↓
Overweight/Obesity ^h^	[333]	8 case-control 6 cohort design 8 case-control ^h^ 6 cohort design ^h^	1.18 (0.96–1.45) 1.47 (1.22–1.78) 2.06 (1.61–2.63) 3.11 (2.62–3.69)	No effect ↑ ↑ ↑
Physical activity	[334]	14 case-control 19 cohort design	RR 0.72 (0.64–0.80) RR 0.84 (0.78–0.91)	↓ ↓
Red Meat	[196]	7 case-control	1.60 (1.26–2.03)	↑
Saturated fatty acids	[335]	10 case-control 4 cohort design	RR 1.06 (0.98–1.14) RR 0.97 (0.93–1.00)	No effect No effect
Selenium	[336] [337]	1 case-control 1 cohort design	0.74 (0.47–1.17) 0.90 (0.53–1.50)	No effect ↓
Soy isoflavones	[329]	5 case-control 2 prospective	0.82 (0.69, 0.97) 0.80 (0.51, 1.26)	↓ No effect
Vitamin A ^i^	[220]	12 case-control 1 cohort	0.88 (0.79–0.98) 0.94 (0.84–1.05)	↓ No effect
Vitamin C	[220]	12 case-control 1 cohort	0.85 (0.73–0.98) 1.01 (0.79–1.28)	↓ No effect
Vitamin D	[220]	3 case-control	0.85 (0.34–2.13)	No effect
Vitamin E	[220]	12 case-control 1 cohort	0.91 (0.84–0.99) 1.06 (0.82–1.37)	↓ No effect

^a^ Women who drank <13 g of alcohol per day had a small reduced risk 0.96 (0.93–1.00). ^b^ Includes other non-steroidal anti-inflammatory drugs; ≥2 aspirin tablets per week. ^c^ Effect of weight reduction intervention for women already diagnosed with EC. ^d^ Dietary cadmium supplementation. ^e^ Caffeinated coffee; ^f^ De-caffeinated coffee. ^g^ Calculated as the mean difference in fasting insulin levels. ^h^ Defined as BMI ≥30 kg/m^2^. ^i^ Vitamin A including β carotenoids. Abbreviations: RR = relative risk. ↑ = increased effect. ↓ = decreased effect.

**Table 3:** Major driver genes used in the classification of endometrial carcinomas and their somatic frequencies.

Somatic Change	Gene Product and Function	Classification Frequencies *
Mutated *PTEN*	Phosphatase And Tensin Homolog Tumour suppressor gene that antagonises the PI3K-AKT/PKB signalling pathway	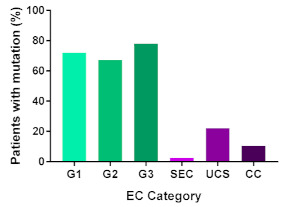
Mutated *ARID1A*	AT-Rich Interactive Domain-Containing Protein 1A A component of SWI/SNF chromatin remodelling complexes that represses expression of select genes through changing chromatin structure	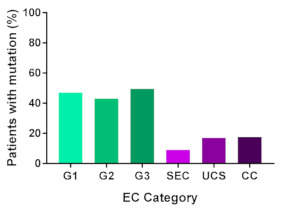
Mutated *PIK3CA*	Phosphatidylinositol-4,5-Bisphosphate 3-Kinase Catalytic Subunit Alpha Participates in cellular signalling in response to various growth factors and is involved in the activation of AKT1-PDPK1 signalling cascades involved in cell growth and survival.	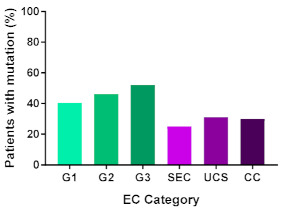
Mutated *PIK3R1*	Phosphoinositide-3-Kinase Regulatory Subunit 1 Acts as an adapter, mediating the association of the p110 catalytic unit to the plasma membrane, which is necessary for the insulin-stimulated increase in glucose uptake and glycogen synthesis in insulin-sensitive tissues.	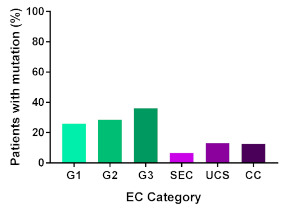
Mutated *KRAS*	KRAS Proto-Oncogene, GTPase Plays an important role in the regulation of cell proliferation by promoting oncogenic events by inducing transcriptional silencing of tumour suppressor genes.	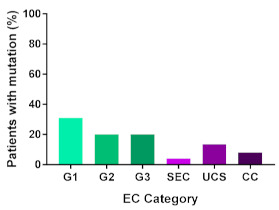
Mutated *CTNNB1*	Catenin Beta 1 A key component in the Wnt signalling pathway and in insulin internalisation. When mutated, it accumulates in the nucleus, where it acts as a coactivator for transcription factors of the TCF/LEF family, leading to activation of Wnt responsive genes.	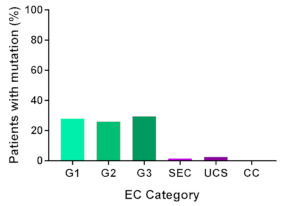
Microsatellite Instability (MSI)	This genetic aberration is associated with dysfunctional DNA mismatch repair mechanisms. The major protein product MLH1 interacts with PMS2 to introduce single-strand breaks near the DNA mismatch and thus generates new entry points for the exonuclease EXO1 to degrade the strand containing the mismatch.	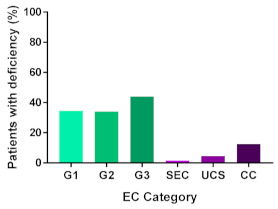
Mutated *FGFR2*	Fibroblast Growth Factor Receptor 2 Plays an essential role in the regulation of cell proliferation, differentiation, migration and apoptosis and mediates activation of RAS, MAPK1/ERK2, MAPK3/ERK1 and the MAP kinase signalling pathway, as well as the AKT1 signalling pathway.	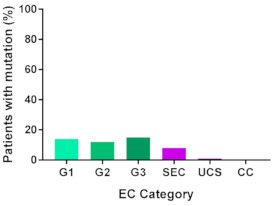
Mutated *POLE*	DNA Polymerase Epsilon, Catalytic Subunit Catalytic component of the DNA polymerase epsilon complex and participates in chromosomal DNA replication. It has 3’-5’ proofreading exonuclease activity that corrects errors arising during DNA replication and so aids DNA synthesis during DNA repair.	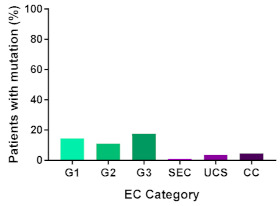
Mutated *TP53*	Tumor Protein P53 Acts as a tumour suppressor in many tumour types; induces growth arrest or apoptosis depending on the physiological circumstances and cell type. Apoptosis induction is mediated either by stimulation of BAX and FAS antigen expression, or by repression of Bcl-2 expression.	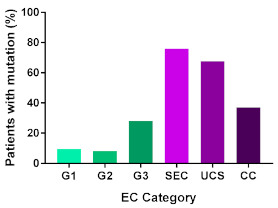
Mutated *FBWX7*	F-Box And WD Repeat Domain Containing 7 Substrate recognition component of a SCF (SKP1-CUL1-F-box protein) E3 ubiquitin-protein ligase complex which mediates the ubiquitination and subsequent proteasomal degradation of target proteins involved in cell cycle control.	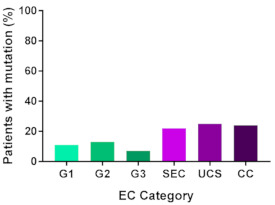
Mutated *PPP2R1A*	Protein Phosphatase 2 Scaffold Subunit A alpha Required for proper chromosome segregation and for centromeric localisation of shugoshin 1 (SGO1) in mitosis, resulting in increased cell proliferation.	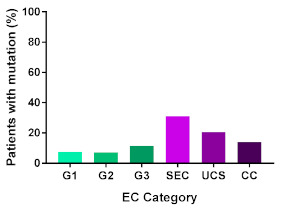
Amplified *ERBB2*	Erb-B2 Receptor Tyrosine Kinase 2 In the nucleus, this protein inhibits the transcription of Cyclin Dependent Kinase Inhibitor 1A (CDKN1A), a key regulator of the cell cycle and TP53 mediated inhibition of cellular proliferation in response to DNA damage.	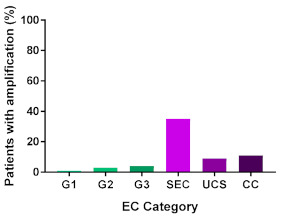

* Type 1 EC are shown in green bars and Type 2 EC are shown in purple bars. G = grade of tumour; SEC = serous EC; UCS = uterine carcinosarcoma; CC = clear cell carcinoma.
